# The Promise of Sleep: A Multi-Sensor Approach for Accurate Sleep Stage Detection Using the Oura Ring

**DOI:** 10.3390/s21134302

**Published:** 2021-06-23

**Authors:** Marco Altini, Hannu Kinnunen

**Affiliations:** 1 Oura Health, Elektroniikkatie 10, 90590 Oulu, Finland; hannukin@gmail.com; 2 Department of Human Movement Sciences, Vrije Universiteit Amsterdam, De Boelelaan 1105, 1081 HV Amsterdam, The Netherlands

**Keywords:** sleep staging, wearables, heart rate variability, accelerometer, machine learning

## Abstract

Consumer-grade sleep trackers represent a promising tool for large scale studies and health management. However, the potential and limitations of these devices remain less well quantified. Addressing this issue, we aim at providing a comprehensive analysis of the impact of accelerometer, autonomic nervous system (ANS)-mediated peripheral signals, and circadian features for sleep stage detection on a large dataset. Four hundred and forty nights from 106 individuals, for a total of 3444 h of combined polysomnography (PSG) and physiological data from a wearable ring, were acquired. Features were extracted to investigate the relative impact of different data streams on 2-stage (sleep and wake) and 4-stage classification accuracy (light NREM sleep, deep NREM sleep, REM sleep, and wake). Machine learning models were evaluated using a 5-fold cross-validation and a standardized framework for sleep stage classification assessment. Accuracy for 2-stage detection (sleep, wake) was 94% for a simple accelerometer-based model and 96% for a full model that included ANS-derived and circadian features. Accuracy for 4-stage detection was 57% for the accelerometer-based model and 79% when including ANS-derived and circadian features. Combining the compact form factor of a finger ring, multidimensional biometric sensory streams, and machine learning, high accuracy wake-sleep detection and sleep staging can be accomplished.

## 1. Introduction

An increasing proportion of the population is tracking their health using wearable technology, with sleep being a prime parameter of interest. Part of the motivation behind tracking sleep is due to the recognition of sleep as an essential aspect of physical health (e.g., weight control, immune health, blood-sugar regulation) [1,2,3], as well as of mental and cognitive brain health (e.g., learning, memory, concentration, productivity mood, anxiety, depression) [4,5]. As such, wearable devices offer the promise of a daily feedback tool guiding personal health insights and, thus, behavioral change that could contribute to a longer healthspan and lifespan. However, for wearable devices to become broadly adopted, the correct form-factor becomes key to maintain adherence [6,7]. This is similarly true of the utility of the type and accuracy of sensory data that such devices provide to the user, and whether that data is real-world meaningful to them [8,9].

Beyond adoption of sleep trackers by the general public, there is also growing interest from academic researchers and clinicians to better understand how to utilize sleep tracking data from consumer devices [6,10]. Early evidence indicated that consumer-grade sleep trackers have promise as tools for large scale sleep studies [11,12]. Building on this, scientists and clinicians are seeking to understand the accuracy of these new technologies, relative to gold-standard measures of sleep, such as polysomnography (PSG). This sentiment has recently been reaffirmed by the American Academy of Sleep Medicine (AASM) in their position statement regarding commercial sleep trackers [13].

The gold-standard for measuring sleep is PSG, a comprehensive, multi-parameter test that is usually performed in a sleep lab. PSG typically records brain wave signals (EEG), eye movement signals (EOG), cardiac signals (ECG), muscle activity (EMG), and, optionally, finger photoplethysmography (PPG). Using this combination of data, human experts or algorithms can determine the different stages of sleep (N1, N2, N3, REM, and wake) across the night, a process referred to as sleep staging. The classification is possible due to specific electrophysiological patterns present in each sleep stage, and recorded by the EEG, EOG, and EMG data. For example, EOG is particularly useful when identifying REM sleep, due to the rapid eye movement which defines this sleep stage. On the other hand, deep sleep is characterized by large amplitude slow-waves visible in EEG data [14,15]. Sleep staging is then performed in successive segments of 30 s and according to the standardized rules of the AASM. While PSG is the gold-standard, the overall inter-scorer reliability for sleep staging has been reported to be only 82–83% [16], with the weakest reliability found for N1, a transition stage between wakefulness and sleep. N1 is, however, usually combined with N2 sleep in wearable devices. The combination of N1 and N2 is called light sleep to differentiate them from the deepest sleep stage, N3 sleep.

In addition to PSG, actigraphy has been a widely used research-grade method for sleep-wake assessment. However, actigraphy has limitations in quantifying other features of sleep. When compared to PSG for sleep assessment in healthy subjects, actigraphy has an overall sensitivity range of 72–97%, specificity range of 28–67%, Pearson’s correlation coefficients for total sleep time (TST) of 0.43–0.97, sleep onset latency (SOL) of 0.64–0.82, and wake after sleep onset (WASO) of 0.36–0.39 [17]. Although actigraphy has proven to be helpful for basic wake-sleep assessment, alone, it has a limited accuracy, especially regarding the differentiation of NREM and REM sleep stages [18,19].

In contrast, when actigraphy is combined with measures of the autonomic nervous system (ANS) in wearables, the accuracy of sleep quality estimations relative to PSG is equivalent to consumer EEG devices in terms of sleep-wake assessment [18,20,21]. In the past decade, several devices and algorithms have been developed to investigate the relation between ANS activity and sleep stages. Additionally, various consumer technology devices have been brought to market utilizing a combination of accelerometer and physiological data. For example, the first generation Oura ring had a 96% sensitivity to detect sleep, and agreement of 65% for light sleep, 51% for deep sleep, and 61% for REM sleep in adolescents and young adults [11]. In another study on Fitbit devices, the estimated Cohen’s kappa was 0.52±0.15 and an epoch by epoch accuracy of 69% was reported [22]. NREM–REM classification of the Emfit bed sensor (based on ballistocardiography) achieved a total accuracy of 79±9%, but a kappa of only 0.43±0.17, highlighting how accuracy is not an ideal metric for a classification problem with highly imbalanced data [23]. In general, the more brain states (e.g., NREM, REM, wake, sleep) that a classifier aims to define, the harder it is to achieve higher accuracy [20,21,24].

Field evaluation of sleep quality has improved by miniaturized sensor technology and superior mathematical modeling. For 4-class sleep stage classifications, this is especially true when based on multidimensional sensor streams combining accelerometer and ANS data, together with machine learning approaches. Cohen’s kappa for actigraphy alone is typically reported at 0.5, while including ANS features improved results up to kappa = 0.6, with small incremental improvements reported when using band-adapted frequency domain heart rate variability (HRV) features [25]. Furthermore, mathematical modeling of the circadian rhythm, has been used to account for differences in sleep stage frequency across the night; for example, using simply the time elapsed during the night as a feature, the higher relative frequency of REM sleep in the second part of the night can be better accounted for, leading to improved classification accuracy [26].

While making significant advancement, to date, these results have limitations. First, a limited amount of sleep data was typically collected and analyzed in a local setting, limiting accuracy confidence and generalizability. By including a larger number of participants with a diverse background (age, ethnicity, sex, etc.), physiological and behavioral patterns could be better captured and, therefore, improve model performance on unseen data. Second, there has been limited information concerning how different sensor data and circadian sleep models contribute to sleep quality evaluations in globally distributed data. Third, the benefit of ANS-mediated peripheral signals available in wearable devices for the assessment of sleep quality has not been clearly quantified, for a number of reasons. This includes measures of the ANS from lower quality sources that are subjected to error distortion, as can happen from the wrist or arm [27,28,29]. Fourth, while it is clear from published literature that accelerometer, ANS, temperature, and circadian rhythm-derived features are all discriminative of different physiological changes occurring during sleep, no comprehensive and systematic analysis of the relative impact of these features has been reported on a large set of individuals. Finally, sleep staging results from different studies are unfortunately not directly comparable due to differences in the study population, sleep staging, data quality, and data processing techniques.

Building on these advancements and limitations, and seeking to move closer to the goals set forth by the AASM guidelines [13], here, we sought to collect a wide dataset in order to test the hypothesis that a combined approach would result in improved performance, approaching results shown by EEG-based systems. We show that the proposed approach, including accelerometer, temperature, heart rate, HRV features, and mathematical modeling of the circadian rhythm, together with features normalization and machine learning techniques, outperforms current state of the art during cross-validation for 4-stage classification.

## 2. Materials and Methods

To investigate the relative contribution of different feature sets on sleep staging classification accuracy, and evaluate the robustness of the proposed model in different populations, we compare four models, described below. Additionally, we evaluate the four models on a large dataset comprising accelerometer, physiological data, and reference PSG collected in three continents. Specifically, we collected data on 440 nights and 106 participants, for a total of 3444 h on three continents (Asia, Europe, and North America). Data was acquired using a research version of the Oura ring able to store in full resolution accelerometer, temperature, heart rate, and HRV, as described below. The four models developed include the following feature sets: accelerometer (ACC) accelerometer and temperature (ACC+T), accelerometer, temperature, and HRV (ACC+T+HRV), and, finally, accelerometer, temperature, HRV, and circadian features (ACC+T+HRV+C). We report results for 2-stage (wake/sleep) and 4-stage (wake, light, deep, REM) classification using a standardized framework for testing performance of sleep tracking technology [30].

### 2.1. Datasets and Data Acquisition Protocols

**Figure 1:**
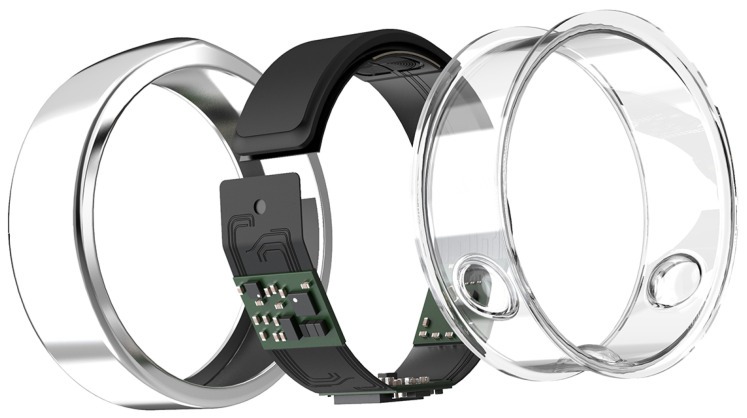
Technical illustration of the second generation Oura ring. The ring has a titanium cover, battery, power handling circuit, double core processor, memory, two LEDs, a photosensor, temperature sensors, 3-D accelerometer, and Bluetooth connectivity to a smartphone app.

For the purpose of model development and validation, we acquired three datasets, in Singapore, Finland, and the United States of America (USA). Each dataset includes data collected with a reference PSG device and the research version of the Oura ring. More details on the PSG device used for each individual study are reported below. The Oura ring is a consumer-based health tracking device which measures body signals, such as heart rate, HRV, respiration, body temperature, and movement, via infrared photoplethysmography (PPG), negative temperature coefficient (NTC), and a 3-D accelerometer. All sensors are located on the inside of the ring, lying adjacent to the underside (palm side) of the finger. The device is powered by a USB charging base. The Oura Ring is water-resistant up to 100 m. Thus, the participant can bathe, swim, wash dishes, and carry out most activities that involve water. The model used in this study is the “Gen2M”, which contains identical sensors and additional flash memory to the commercially available Oura Ring, shown in Figure 1.

During data collection, the start and stop times were decided based on the PSG lights off and lights on labels, manually annotated by the participants (for home-based measurements) or experimenters (for lab-based measurements). The Oura ring comes in different sizes, which were selected on a participant by participant basis depending on personal comfort. In particular, for Dataset 1: Singapore, participants wore the Oura ring on the hand in which they could achieve the best fit with the sizes available. For Dataset 2: Finland, data was collected from both hands but algorithm development included only data from the left hand not to duplicate entries. Finally, for Dataset 3: USA, data was collected from both hands but algorithm development included only data from the right hand. By including data collected from both hands from different individuals, we aimed at developing an algorithm that could perform well independently of sensor location. To ensure high data quality, participants were instructed to position the LEDs of the ring on the bottom side of their fingers when going to bed. Due to proper ring selection, and the typical limited movement during night, together with the fact that fingers tend to get swollen during sleep, the ring typically does not move or rotate during the night, ensuring high data quality. The data collection protocol was designed to resemble real life scenarios; therefore, no extra measures, like taping the ring, were taken.

### 2.2. Participants

#### 2.2.1. Dataset 1: Singapore

Fifty-nine healthy adolescents (30 female), age range between 15 and 19 years old, mean 16.4±1.1 years old, participated in this study, each participant recording up to 8 nights, for a total of 322 recordings. All participants were screened for sleep disorders and pre-existing medical conditions prior to the study, had body mass index (BMI) of less than 30 kg/m${}^{2}$, and did not smoke. PSG was acquired using the SOMNOtouch device (SOMNOmedics GmbH, Randersacker, Germany). Sleep scoring was performed with the validated Z3Score algorithm [31] and visually checked by trained technicians who were blinded to the Oura ring records. Wake, REM sleep, N1, N2, and N3 sleep were calculated based on 30-s epochs according to the standard criteria of the AASM. More details on the protocol for this study can be found in References [31,32]. The Institutional Review Board of the National University of Singapore approved the study, which was in accordance with the principles in the Declaration of Helsinki. Informed written consent was obtained from participants and their legal guardians.

#### 2.2.2. Dataset 2: Finland

Nineteen healthy adults (11 female), age range between 24 and 51 years old, mean 38.6±8.5 years old, participated in this study, for a total of 21 nightly recordings. Participants were recruited from the Oulu region in Finland. In the afternoon prior to the PSG recording, subjects were informed orally about the study procedure and they were advised about their right to withdraw from the investigation at any time. Participants gave a written informed consent to participate. Three participants volunteered to participate for two nights. A certified sleep technologist from the Finnish Institute of Occupational Health set up PSG sensors (Embla Titanium, Natus Medical Inc., Pleasanton, CA, USA) before subjects left for a home measurement. Sleep scoring was performed by the same sleep technologist, who was blinded to the Oura ring records. All participants were apparently healthy as none of the subjects reported any diagnosed sleep issue, health condition, or medication that was thought to affect sleep.

#### 2.2.3. Dataset 3: USA

Forty generally-healthy adults (24 female), age range between 20 and 73 years old, mean 45.0±15.2 years old, participated in this study, for up to 7 nights each, leading to a total of 191 nightly recordings. Participants were recruited using SleepMed’s research database of healthy participants. No evidence of sleep pathology was reported. Reference PSG was collected using the A1 PSG system manufactured by NOX Medical. Informed consent was obtained prior to data collection and the study was approved by the local institutional review board. PSG was visually scored by SleepMed’s team of registered sleep technologists on a 30 s epoch basis for sleep stages, blinded to the Oura ring records.

Following removal of entire nights in which technical errors occurred, 440 nights from 106 participants were used for data analysis, for a total of 3444 h. The overall age range was between 15 and 73 years old, with a mean of 30.1±16.4 years old.

### 2.3. Features Extraction

Features were extracted offline from the available data streams (accelerometer, PPG, and temperature) using sliding windows of different lengths based on the relation between these data streams and sleep stages. For example, window lengths of 1 and 5 min were used for HRV analysis to capture both short-term or faster changes in parasympathetic activity, as well as longer-term changes, as typically present in resting heart rate. When considering multiple features associated with similar physiological mechanisms, we prioritized features with lower computational complexity, to enable faster execution time and potentially ease a future embedded implementation in real-time. More details on each data stream are provided below. Additionally, we included sensor-independent features representative of the circadian rhythm which have shown to improve sleep stage classification in previous research [26].

#### 2.3.1. Accelerometer Data

The Oura ring includes a triaxial accelerometer which was configured to record data at a sampling frequency of 50 Hz and a resolution of ±2 g. First, standard descriptive statistics were calculated on each individual axis, after applying a 5th order butterworth bandpass-filter between 3 to 11 Hz and taking the absolute of the filtered values [33]. The features include the trimmed mean (after removing 10% on each side), max, and interquartile range (IQR) of each axis, calculated in successive windows of 30 s. Second, the mean amplitude deviation (MAD [34]) is calculated in epochs of 5 s from the unfiltered accelerometer data. The MAD is based on the deviation from the vector magnitude of the current 5-s epoch. For each 30-s epoch, the trimmed mean, max, and IQR of the MAD are calculated. Third, the difference in arm angle was calculated in 5-s epochs [35], and then aggregated in 30-s epochs using the trimmed mean, max, and IQR.

#### 2.3.2. Temperature Data

**Figure 2:**
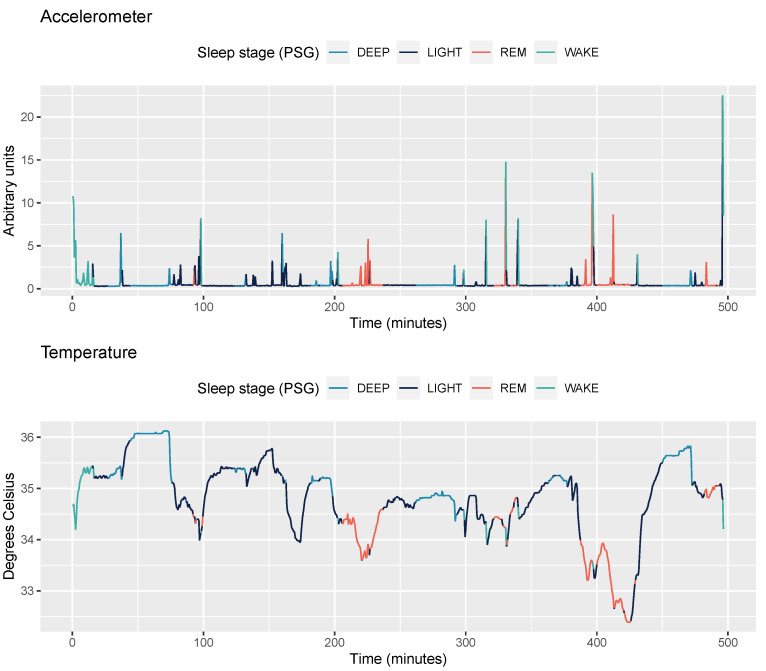
Accelerometer and temperature data for one participant (Dataset 1: Singapore, 15 years old) and one night. Sleep stages annotated from PSG data are color-coded.

The Oura ring has a negative temperature coefficient (NTC) thermistor (non-calibrated, resolution of 0.07 degrees Celsius) as its temperature sensor. The sensor has been programmed to register skin temperature readings from the palm side of the finger base every 10 s. First, we aggregated temperature data into epochs of 30 s, to be consistent with sleep staging. Second, an artifact rejection step is applied, in which values outside the range of 31–40 degrees Celsius are masked. Third, the following features are calculated: mean, min, max, and standard deviation. An example accelerometer and temperature features are shown in Figure 2.

#### 2.3.3. PPG data

**Figure 3:**
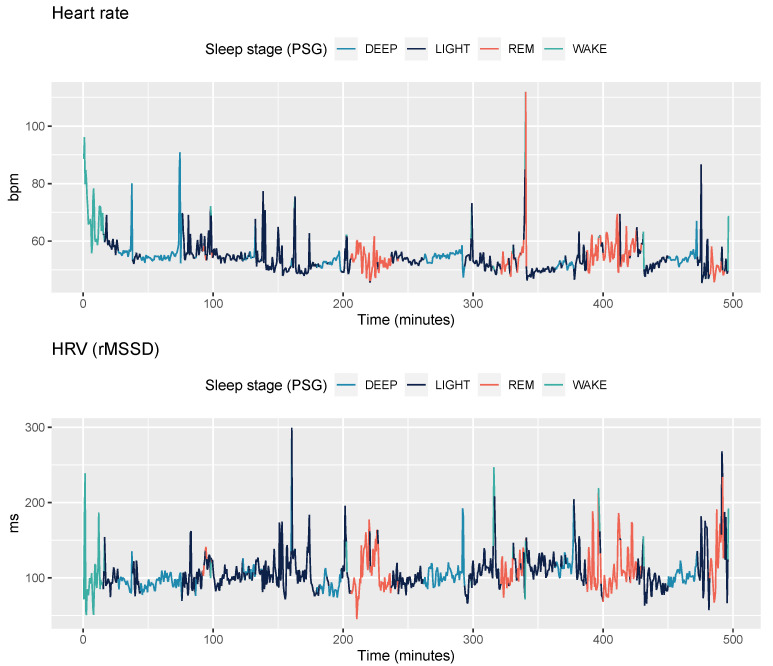
Heart rate and HRV (rMSSD) data for one participant (Dataset 1: Singapore, 15 years old) and one night. Sleep stages annotated from PSG data are color-coded.

To compute ANS-derived features, such as heart rate and HRV, we first processed raw PPG collected from the Oura ring at 125 Hz. In the Oura ring, the photodetector is positioned in the down middle while the two infrared light LEDs (900 nm) are positioned within a short distance on the left and right side of this photodetector. Thus, the measurement is partially transmissive and partially reflective. To derive beat to beat data used to compute HRV features, a real-time moving average filter is applied to locate local maximum and minimum values that denote the timing of each heartbeat. This procedure allows to identify artifacts by labeling each individual interval as normal or abnormal using median filters. In particular, a deviation by more than 16 bpm from the 7-point median interval duration in its immediate neighborhood is marked as abnormal and discarded [36]. Secondly, an interval is included for further analysis only if five consecutive intervals values are labeled as normal, i.e., two before and two after each are acceptable intervals. Once high quality intervals have been identified according to this procedure, time and frequency domain HRV features were extracted. In particular, we extracted heart rate, rMSSD, SDNN, pNN50, frequency power in the LF and HF bands, the main frequency peak in the LF and HF bands, total power, normalized power, and breathing rate. The motivation behind these particular spectral divisions is the notion that various physiological mechanisms related to HRV manifest themselves within the boundaries of these bands. For instance, vagal activity has been found to be a major contributor to the spectral power in the HF band between 0.15 Hz and 0.4 Hz. The physiological interpretation of the spectral power in the LF band of 0.04 to 0.15 Hz is less certain, with findings attributing influences from both the sympathetic and parasympathetic branches. We also implemented the mean and coefficient of variation of the zero-crossing interval [37]. An example of heart rate and HRV during the night for one participant is shown in Figure 3.

#### 2.3.4. Sensor-Independent Circadian Features

**Figure 4:**
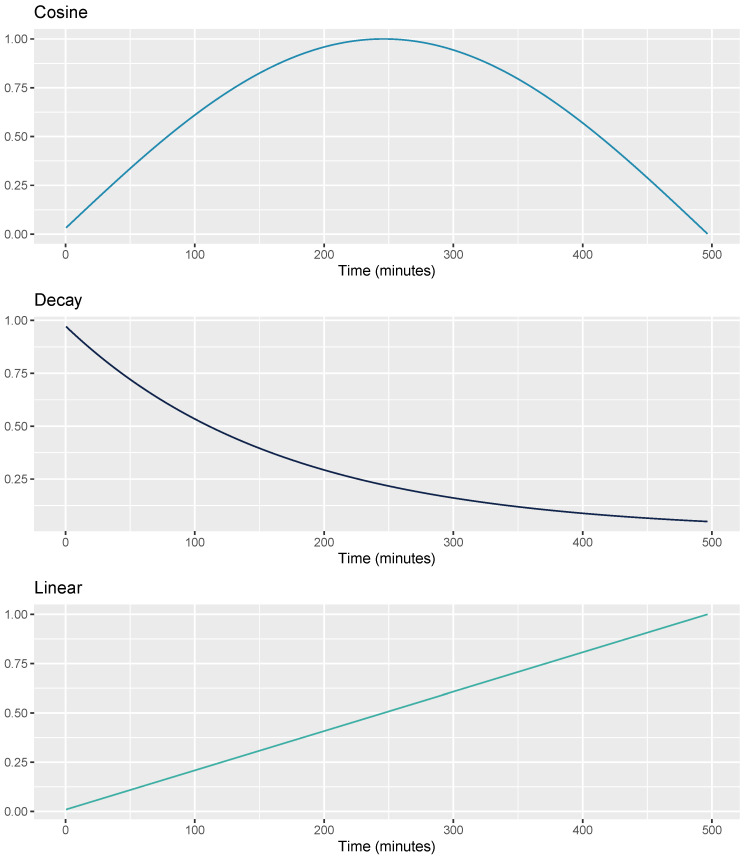
Cosine, decay, and linear functions used to model sensor-independent circadian features.

Sleep is a dynamic process regulated by many internal and external factors. According to the traditional two-process model of sleep [38,39], there are two main components that determine the time when we go to sleep and the time when we wake up, as well as the overall structure and depth of our sleep. These two components are the circadian rhythm and homeostatic sleep drive. The circadian rhythm promotes sleep at night and wakefulness during the daytime. This wave-like rhythm has an internal 24-h period, which is synchronized by external timing cues, such as sunlight. The homeostatic sleep drive refers to how the pressure for sleep linearly builds up in our brain during wakefulness, and decreases in an exponential manner during sleep, and especially deep NREM sleep. We have added three sensor-independent features to capture these two components, a cosine function, an exponential decay, and a linear function. The cosine function represents the circadian drive component [26,40]. The second feature represents the decay of homeostatic sleep pressure across the night, most dramatically during the first hours of sleep which are rich in deep NREM sleep. The third feature is the time elapsed since the beginning of the night, as a linear function ranging from 0 to 1. The goal of this feature is to take into account the well-known asymmetry of sleep stages across a typical night of sleep (i.e., more deep NREM early in the night, and more REM sleep in the last hours of the night). Sensor-independent features were computed using as parameter the duration of each individual night of sleep, as sleep staging is performed only at the end of the night. An example of these three features is shown in Figure 4.

### 2.4. Feature Normalization

The physiological principle behind using ANS activity for sleep stage classification is that there are quite large differences in sympathetic and parasympathetic activity across sleep stages, and these differences can be captured as relative changes over time, within individuals. However, while all features included in our analysis have some discriminatory power to detect different sleep stages, physiological measurements are highly individual, and absolute values can differ greatly between individuals based on parameters other than the ones of interest (e.g., genetics, age, etc.). Thus, we normalized features on a per-night basis using a robust method based on the 5–95 percentiles [37]. This step is critical to account for inter-individual differences in features (e.g., nightly heart rate or HRV). A few features, mostly from the accelerometer, were not normalized as they provide information about the absolute magnitude of movement, which is useful to detect short awakenings during the night.

### 2.5. Machine Learning

Model training and testing was performed using a Light Gradient Boosting Machine (LightGBM [41]) classifier, with a DART [42] boosting and 500 estimators. LightGBM typically provides high accuracy, fast training, low memory usage, and is capable of handling missing values at instances where data is of too poor quality to calculate features.

### 2.6. Validation Strategy and Evaluation Metrics

Machine learning models were evaluated using a 5-fold cross-validation and a standardized framework for sleep stage classification assessment [30]. According to the proposed framework, we report for all models and both 2-stage and 4-stage classification the time spent or estimated in each sleep stage, as well as Bland-Altman plots. We also report sensitivity and specificity for epoch by epoch analysis of each individual sleep stage. In addition to the standardized framework, we report the f1 score as an additional performance metric. The f1 score provides a better overview of the classifier performance in multiclass classification with imbalanced data, which is the case for sleep stage detection (i.e., light sleep is far more frequent than deep sleep, REM sleep, or wake). All analyses were performed in Python 3.8 using the pandas, numPy, scikit-learn, and seaborn packages. In addition, the R script provided in Reference [30] was used to generate Bland-Altman plots and other results according to the standardized validation framework.

## 3. Results

### 3.1. 2-Stage Classification

**Figure 5:**
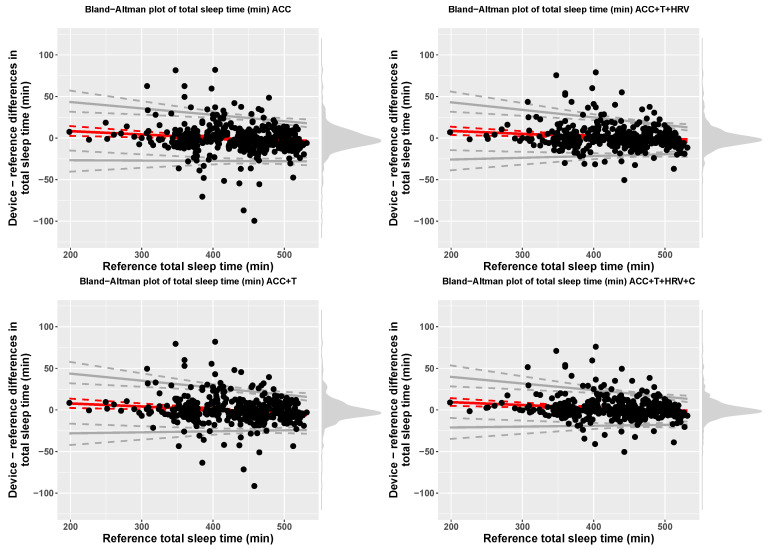
Bland-Altman plots for total sleep time (TST), 2-stage classification, and the four models compared in this paper.

Accuracy for 2-stage detection (sleep, wake) was 94% (f1-score = 0.67), for accelerometer-based models, while including temperature resulted in 95% accuracy (f1-score = 0.69), including HRV features resulted in 96% accuracy (f1-score = 0.73), and, finally, adding circadian features, led to 96% accuracy (f1-score = 0.78). At the group level, Table 1 reports total sleep time (TST) for each model, including bias and limits of agreement, while Bland-Altman plots of the four models are shown in Figure 5.

**Figure 6:**
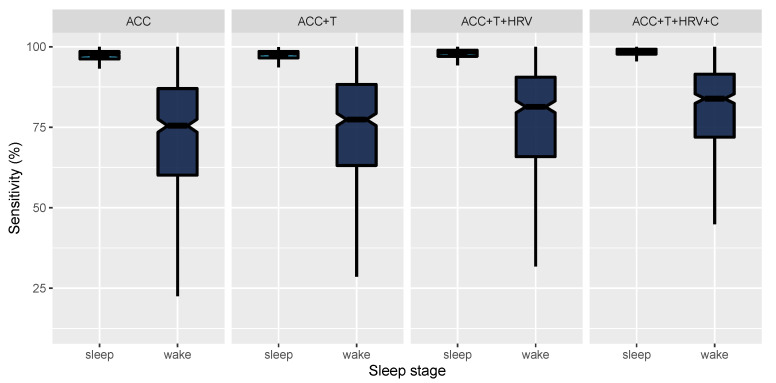
Epoch by epoch sensitivity for sleep and wake and the four models compared in this paper. Whiskers are computed as 1.5 times the interquartile range.

In Figure 6, we report epoch by epoch sensitivity for sleep and wake and the four models compared in this paper. Sensitivity is also reported in Table 2. Since we are dealing with 2-stage classification, specificity for wake is the same as sensitivity for sleep and, therefore, is not reported separately.

### 3.2. 4-Stage Classification

**Figure 7:**
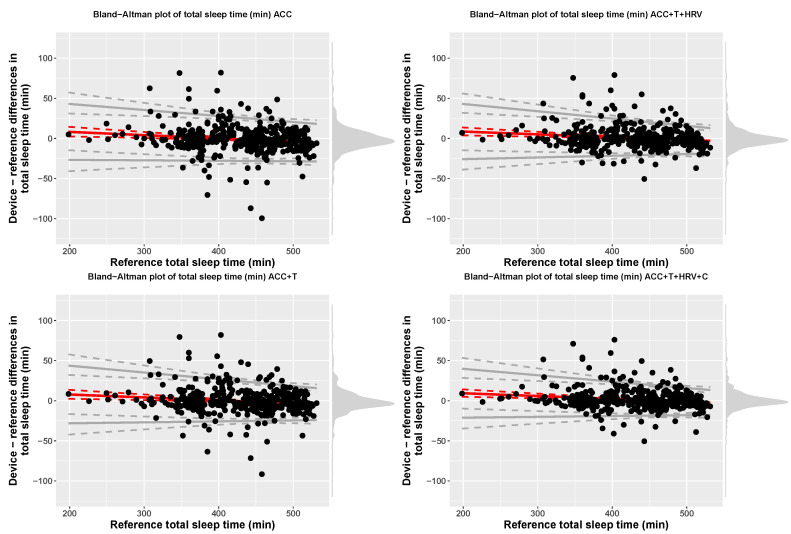
Bias and limits of agreement for TST, 4-stage classification, and the four models analyzed in this paper.

**Figure 8:**
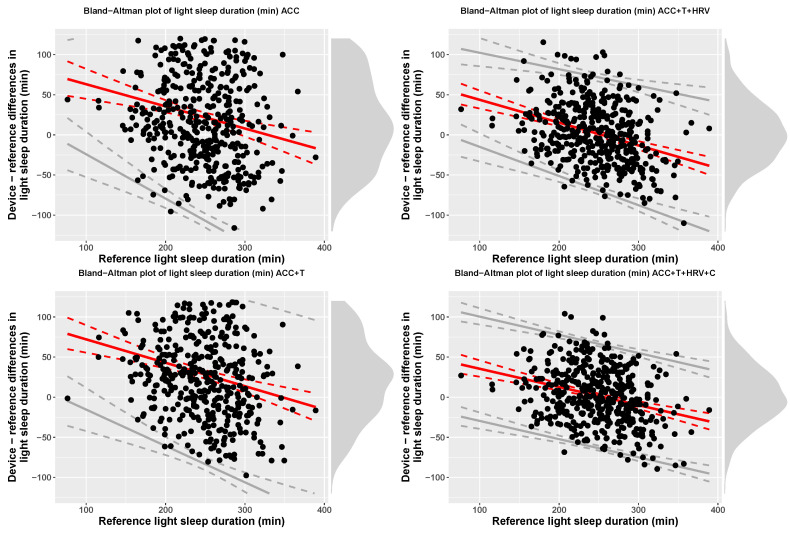
Bias and limits of agreement for time in light sleep, 4-stage classification, and the four models analyzed in this paper.

**Figure 9:**
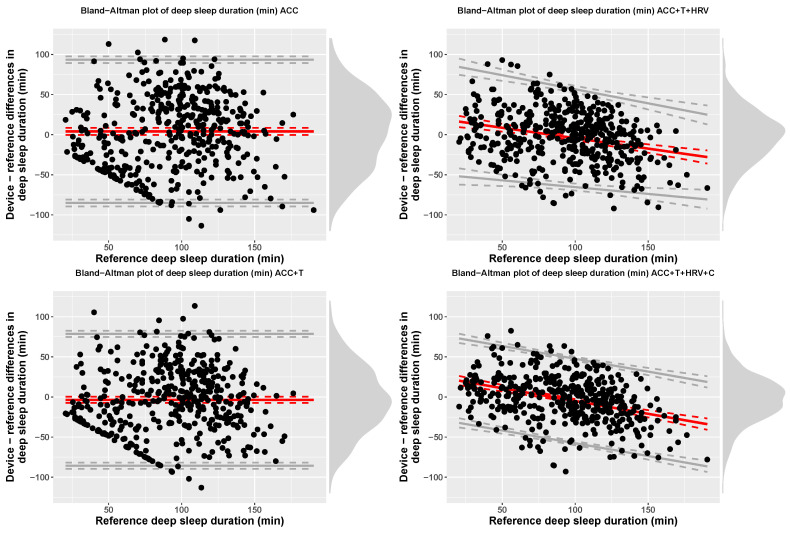
Bias and limits of agreement for time in deep sleep, 4-stage classification, and the four models analyzed in this paper.

**Figure 10:**
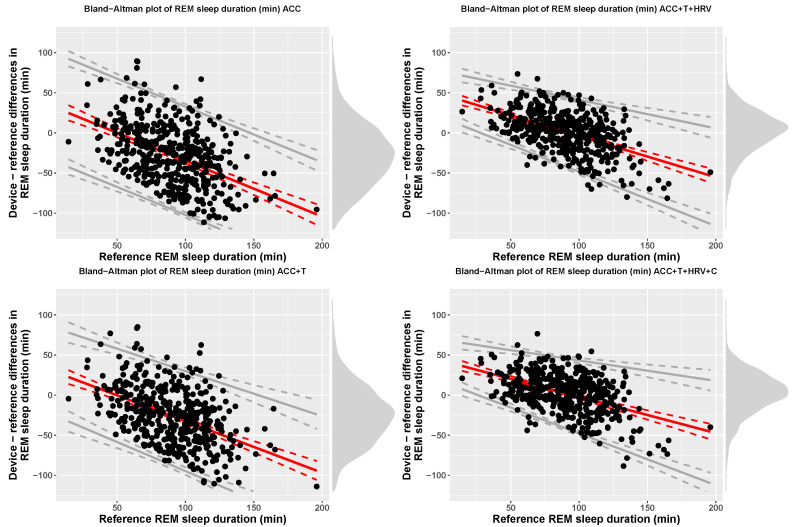
Bias and limits of agreement for time in REM sleep, 4-stage classification, and the four models analyzed in this paper.

Accuracy for 4-stage detection (sleep, wake) was 57% (f1-score = 0.68), for accelerometer-based models, while including temperature resulted in 60% accuracy (f1-score = 0.69), including HRV features resulted in 76% accuracy (f1-score = 0.73), and, finally, adding circadian features, led to 79% accuracy (f1-score = 0.78). At the group level, Table 3 reports TST, time in light, deep, and REM sleep for each model, including bias and limits of agreement. Figure 7, Figure 8, Figure 9 and Figure 10 show Bland-Altman plots of the four models for TST, light, deep, and REM sleep.

**Figure 11:**
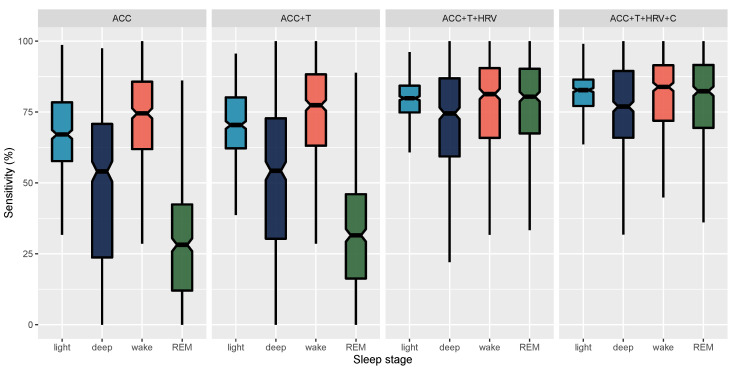
Epoch by epoch sensitivity for 4-stage classification and the four models compared in this paper. Whiskers are computed as 1.5 times the interquartile range.

**Figure 12:**
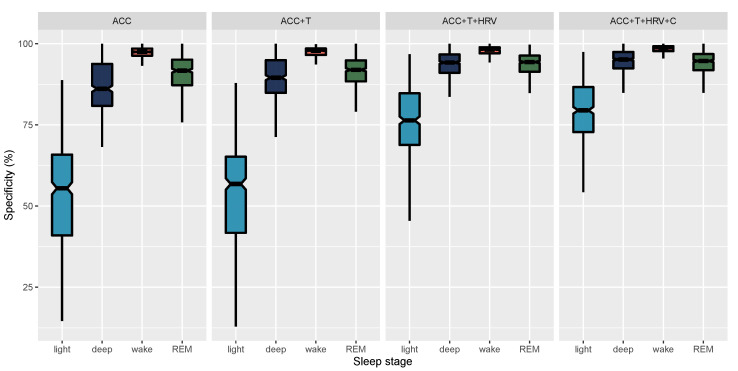
Epoch by epoch specificity for 4-stage classification and the four models compared in this paper. Whiskers are computed as 1.5 times the interquartile range.

In Figure 11, we report epoch by epoch sensitivity, while, in Figure 12, we report epoch by epoch specificity for 4-stage classification and the four models compared in this paper. Sensitivity and specificity are also reported in Table 4.

**Figure 13:**
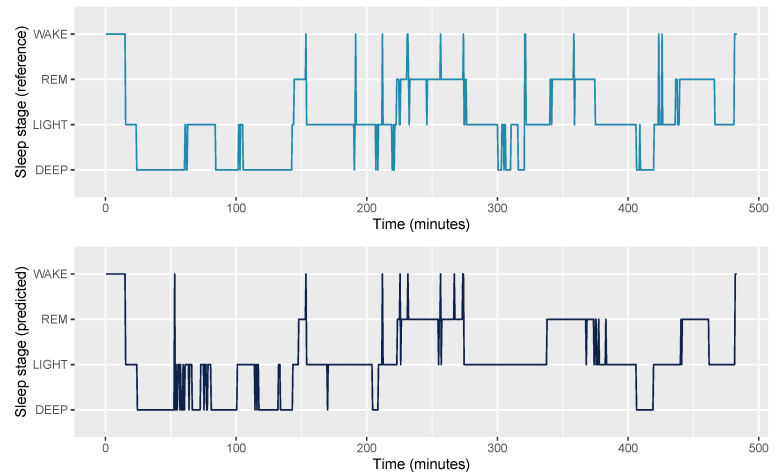
Example hypnogram for an average night (f1 = 0.78) for the model, including all features (ACC+T+HRV+C).

An example hypnogram for an average night (f1 = 0.78) is shown in Figure 13.

## 4. Discussion

Consumer-grade sleep trackers represent a promising tool for large scale studies and health management, yet the potential of these devices remain less well quantified. Addressing this issue, here, we provided a comprehensive analysis of the ability of a wearable device measuring accelerometry, ANS-mediated peripheral signals, and modeling circadian features to detect sleep stages accurately, with respect to gold-standard PSG recordings. The findings demonstrate that robust sensitivity, specificity and accuracy for the staging of wake, light NREM, deep NREM, and REM sleep using a compact, wearable finger ring-format, can be accomplished.

Physiologically, all sleep stages differ from each other in terms of typical breathing, ANS, and body movement patterns. These behavioral differences and physiological responses to sleep stages are driven by a tight coupling between central nervous system activity and ANS activity, which provides the theoretical framework for our work. When combining such data streams from the ring with sensor-independent circadian features designed to better account for differences in sleep stage distribution across the night, as well as features normalization and machine learning techniques, accuracy for 2-stage and 4-stage detection approaches results previously reported only for EEG-based systems [18,43,44].

Importantly, performance of the models developed and implemented in this work were analyzed according to a standardized framework [30]. Specifically, group level performance was analyzed using Bland-Altman plots and reporting bias and limits of agreement for each sleep stage. The results reported in Table 3 show how accelerometer only models can result in a bias up to 30 min for 4-stage detection. On the other hand, combining all data streams and sensor-independent features leads to a very small bias of typically less than 3 min. Similarly, the limits of agreement get narrower as the model includes more features, highlighting improved performance.

When looking at epoch by epoch performance, we can better understand how the different data streams contribute to model performance. In particular, accelerometer-only models can only detect wake, as movement alone cannot differentiate between more complex brain stages. When adding finger temperature, we report small improvements in the detection of different sleep stages. The largest improvement for 4-stage classification performance was obtained when including HRV features, as these are more tightly coupled to brain waves changes occurring during sleep. Adding HRV features provided an improvement in accuracy from 60% to 76% across 4-stages. Notably, adding circadian features that are sensor-independent led to additional improvements in the detection of sleep stages, specifically deep NREM and REM sleep.

One of the main strengths of this work is the high sensitivity and specificity reported across all stages, ranging from 74% to 98%. While other studies have shown similar results for the detection of a specific stage, such as, e.g., deep sleep, this typically comes at the expense of the performance in detecting other sleep stages, e.g., resulting in REM or wake sensitivity as low as 50% [26,45,46]. A significant advance made by this report is the finding that, combining multiple sensor data streams from the finger, as well as circadian-features, and, finally, feature normalization, high sensitivity and specificity for all sleep stages can be accomplished. Building on this recognition, below, we discuss the physiological mechanisms that might be driving this superior stage classification ability.

### 4.1. Physiological Considerations

In terms of accelerometer data, we have designed a set of features that can better discriminate between sleep stages and are less prone to calibration error or hardware differences with respect to motion intensity features typically used in actigraphy. These include capturing relative deviations from previous windows or using trigonometry identities to estimate finger-derived motion in a more robust manner, as these features are less likely to be confounded by, for example, a person’s partner moving in bed. Additionally, features were smoothed using a set of rolling functions, the primary goal of which was to increase sleep staging accuracy by taking into account the past and the future at each epoch. This emulates the way that human scoring experts typically stage sleep, i.e., by constantly keeping track of what happened before the current epoch, as well as what will happen after. While results for accelerometer-only models are still below those of gold standard PSG, especially for 4-stage classification, using the proposed features led to good performance in the detection of sleep stages, including deep NREM sleep that consumer devices have historically struggled to accomplish.

Regarding finger temperature, there is a clear inverse pattern with core core body temperature, so that finger temperature increases across the night and decreases across the daytime [47]. The reason is that core body temperature reductions are mechanistically accomplished through vasodilation of peripheral surface blood vessels of the skin in the extremities, particularly the hands and feet [48]. Thus, temporally, finger temperature precedes core body temperature by 2–3 h, and these changes might be associated with sleep stages, making finger temperature, more so than the wrist or upper arm, particularly optimal for high accuracy sleep onset determination. Relatively, core body temperature follows a 24-h rhythm, with an overall variation of 1C from peak to nadir. Peak temperature occurs in the evening, while the lowest point in temperature occurs at the end of the night. Indeed, sleep onset is more likely to occur when core body temperature is at its steepest rate of decline. Thereafter, core body temperature decreases during NREM sleep, and modestly increases during REM sleep [49,50]. After determination of sleep onset, our findings demonstrate that adding peripheral finger temperature measurement leads to better sleep staging scoring accuracy. However, its contribution to the overall classifier is somewhat negligible when compared to other ring sensor features and streams, such as accelerometer and HRV data. Nevertheless, finger temperature still represents a relevant and important sensory signal to determine sleep onset and offset, making this unique data feature an important and a potentially overlooked one.

The largest improvement in classification performance occured when adding HRV features. Most wearable sensors today use optical technology to capture beat to beat intervals and compute heart rate or more complex HRV features to estimate sleep stages. This is due to the tighter link between central nervous system activity and changes in ANS which can be captured non-invasively using HRV features. In particular, the physiology of sleep [51] shows consistent patterns that are specific to differences between NREM and REM sleep, as well as each individual stage. For example, during REM sleep, heart rate increases and shows higher variability, as visible in Figure 3. Previous work has shown how an improvement of 15–25% in 4-stage classification can be obtained when including heart rate data, with the additional inclusion of HRV features representative of parasympathetic activity leading to increased performance [18,20,21,26]. During NREM sleep, heart rate can progressively decrease [52], a pattern consistent with increased parasympathetic activity. Given the fast nature of these changes which we quantified from the finger pulse waveform, heart rate and HRV may indeed potentially reflect changes in central and autonomic activity which are captured by PSG via brain waves.

The distribution of sleep stages across the night can change due both to idiosyncratic and expected patterns. The latter include both the typical nature of sleep cycles, with stages following a sequence during approximately 70–120 min cycles [53,54], as well as how the distribution of sleep stages changes throughout the night. In particular, deep NREM sleep is typically more present during the first third of the night, while REM sleep is more present during the second half of the night, when each bout of REM can also last longer [51,55]. Modeling the waxing-and-waning of the circadian rhythm across the night, when sleep is the most stable, with core temperature and heart rate close to their minimum diurnal levels, as well as the decay of homeostatic sleep pressure and the time elapsed since the beginning of the night, resulted in improved accuracy up to 79%. Sleep stage detection in literature has tried to account for temporal associations between stages using various techniques, from Markov models to neural networks. However, we show that modeling changes in sleep stage distribution across the night with sensor-independent circadian features provides a clear improvement in classification performance.

Finally, almost all of the features analyzed in this work were normalized per-night using a robust z-score. In other words, the features were expressed as a deviation from the night’s average. This step is crucial to improve the accuracy of sleep staging, mostly because it allows one to take into account the natural variability between participants and to make use of features whose absolute value is typically of little use, given the very large between individual variability (e.g., HRV features).

### 4.2. Generalizability

Automatic sleep stage classification has historically been a challenging problem, wherein reference data is typically suboptimal. This is in part due to the requirement of subjective human application and interpretation of sleep staging rules, which are used by human annotators to determine reference data which is eventually used for sleep stage classification.

In this work, we addressed several limitations of automatic sleep stage detection, resulting in increased accuracy with respect to state of the art for sleep stage detection based on wearable sensors data. First, we collected data on a large set of participants and nights, for a total over 3400 h of recordings. Data was beneficially heterogeneous, as collection was performed in three sites across the globe, with a broad age range. This meant that we could train and evaluate our models on a large sample of participants, which could then better generalize to other populations, outside the limited ones typically considered in small clinical or research studies that aim to validate sleep trackers. For example, only by collecting data in a diverse population can we capture and, therefore, model different sleep patterns, physiological responses to environmental factors (e.g., temperature or humidity), and behavioral factors (e.g., eating habits that could influence sleep), potentially leading to better generalizability of the reported results. Additionally, it is well known that sleep patterns change as we age, with lower amounts of deep sleep typically reported with advancing age. By collecting a large dataset spanning a broad age range (15–73 years old), we could ensure that these patterns were accounted for during model development.

Current scientific literature has helpfully and fruitfully highlighted certain problems with consumer technology products, often related to software updates, black box nature and lack of independent validation. Finding consistent viewpoints on data streams and evaluation strategies could be beneficial since it could speed up the adoption of new technology in research. The best state-of-the-art wearable technology provides access to ANS mediated signals with an unprecedented high wearing comfort and accuracy level [36,56,57]. Wearable devices also provide long-term time series of sleep and ANS mediated signals that have not been available before [58]. Related to these concerns and features, here, we as a wearable company have sought to provide a detailed breakdown of the contribution of different data streams and feature sets for sleep stage classification, as it would seem to serve the research and clinical communities wishing to understand wearable tools, and to aid a similar approach by other wearable companies. Due to the difficulties of comparing individual small sample studies, the analysis reported in this work should provide useful insights on the relative impact of accelerometer, ANS-mediated peripheral signals, and circadian features in the context of both 2-stage and 4-stage classification from a larger, population-based level.

In accordance with the sleep staging rules of the AASM, effective interpretation of sleep staging needs to be validated against and based on different signals (EEG, EOG, and EMG). However, even when using all of these signals as input for sleep staging classification, therefore reducing practical applicability and user comfort, a kappa of 0.68–0.85 for state of the art neural networks models is reported [59]. In situations in which it is not practical to measure brain activity directly, a combination of the data streams utilized in this paper and available via a wearable ring seems optimal.

Given the high sensitivity and specificity for each individual stage, the proposed method might be able to better track individual changes in sleep patterns over time, with respect to currently available sensors and algorithms, which tend to detect accurately only one or two of the four stages [26,45,46].

### 4.3. Limitations

Despite the large sample size, heterogenous in nature and geographical derivation, together with multiple nights per participant and home-based recordings for two of the three datasets collected in this study, our work suffers from several limitations. The first limitation is that ecological validity is limited by wearing reference PSG, which can be a disruption in the participant’s typical sleep patterns. Similarly, data was collected either from the left or right hand based on participants preference or study protocol, but the impact of wearing the Oura ring on a different hand should be investigated in the future. Secondly, data were collected from healthy participants; therefore, our findings cannot be generalized outside of this population to clinical conditions. Finally, while the heterogenous nature of the data is a strength of the proposed approach, data collection carried out in different sites, by different scientists, and using slightly different reference systems might have introduced inconsistencies. However, the high accuracy, low bias, and limited variability in the reported results seem promising.

## 5. Conclusions

Combining accelerometer, temperature, HRV, and circadian features with machine learning techniques and the compact form factor of a finger ring, high-accuracy of wake-sleep detection, as well as sleep staging, can be accomplished. In particular, the accuracy, f1 score, and individual stage sensitivity and specificity reached in this study approaches results typically reported only in EEG-based studies. These data indicate that such a form-factor device, allowing high user compliance and high quality data, offers a promising tool for conducting large scale studies, and, with such data, providing meaningful health management.

## Figures and Tables

**Table 1:** Bias and limits of agreement for total sleep time (TST), 2-stage classification, and the four models analyzed in this paper.

Model	Device	Reference	Bias	LOA.Lower	LOA.Upper
ACC	429.67 (61.05)	430.66 (61.12)	16.38 + −0.04 x ref	bias − 2.46(17.19 + −0.01 x ref)	bias + 2.46(17.19 + −0.01 x ref)
ACC+T	430.1 (61)	430.66 (61.12)	14.98 + −0.04 x ref	bias − 2.46(18.51 + −0.02 x ref)	bias + 2.46(18.51 + −0.02 x ref)
ACC+T+HRV	431.2 (60.48)	430.66 (61.12)	15.49 + −0.03 x ref	bias − 2.46(18.54 + −0.02 x ref)	bias + 2.46(18.54 + −0.02 x ref)
ACC+T+HRV+C	432 (60.34)	430.66 (61.12)	16.27 + −0.03 x ref	bias − 2.46(16.02 + −0.02 x ref)	bias + 2.46(16.02 + −0.02 x ref)

**Table 2:** Epoch by epoch sensitivity for wake and the four models compared in this paper.

Model	Sensitivity	Specificity
ACC	72.08 (18.44) [70.35, 73.86]	96.82 (3.04) [96.54, 97.11]
ACC+T	73.71 (17.9) [72.06, 75.42]	97.05 (2.79) [96.8, 97.31]
ACC+T+HRV	77.18 (16.77) [75.62, 78.76]	97.61 (2.11) [97.42, 97.81]
ACC+T+HRV+C	80.74 (14.12) [79.44, 82.07]	98.15 (1.87) [97.98, 98.33]

**Table 3:** Bias and limits of agreement for TST, time in light, deep, and REM sleep for 4-stage classification and the four models analyzed in this paper.

Model	Measure	Device	Reference	Bias	LOA.Lower	LOA.Upper
ACC	TST (min)	429.49 (61.05)	430.66 (61.12)	16.23 + −0.04 x ref	bias − 2.46(16.96 + −0.01 x ref)	bias + 2.46(16.96 + −0.01 x ref)
ACC	Light (min)	269.78 (71.24)	247.31 (45.3)	90.71 + −0.28 x ref	bias − 2.46(24.26 + 0.11 x ref)	bias + 2.46(24.26 + 0.11 x ref)
ACC	Deep (min)	97.45 (59.24)	93.34 (34.19)	4.11 (45.55)	−85.16	93.38
ACC	REM (min)	62.26 (35.36)	90.01 (26.24)	34.95 + −0.7 x ref	bias − 67.53	bias + 67.53
ACC+T	TST (min)	430.1 (61)	430.66 (61.12)	14.98 + −0.04 x ref	bias − 2.46(18.51 + −0.02 x ref)	bias + 2.46(18.51 + −0.02 x ref)
ACC+T	Light (min)	276.39 (64.67)	247.31 (45.3)	101.27 + −0.29 x ref	bias − 2.46(29.01 + 0.07 x ref)	bias + 2.46(29.01 + 0.07 x ref)
ACC+T	Deep (min)	89.72 (53.45)	93.34 (34.19)	−3.62 (41.93)	−85.8	78.56
ACC+T	REM (min)	63.98 (32.59)	90.01 (26.24)	31.86 + −0.64 x ref	bias − 61.18	bias + 61.18
ACC+T+HRV	TST (min)	431.2 (60.48)	430.66 (61.12)	15.49 + −0.03 x ref	bias − 2.46(18.54 + −0.02 x ref)	bias + 2.46(18.54 + −0.02 x ref)
ACC+T+HRV	Light (min)	249.12 (48.06)	247.31 (45.3)	72.1 + −0.28 x ref	bias − 2.46(20.61 + 0.03 x ref)	bias + 2.46(20.61 + 0.03 x ref)
ACC+T+HRV	Deep (min)	90.69 (40.12)	93.34 (34.19)	21.61 + −0.26 x ref	bias − 2.46(28.47 + −0.04 x ref)	bias + 2.46(28.47 + −0.04 x ref)
ACC+T+HRV	REM (min)	91.39 (25.24)	90.01 (26.24)	47.76 + −0.52 x ref	bias − 2.46(11.74 + 0.07 x ref)	bias + 2.46(11.74 + 0.07 x ref)
ACC+T+HRV+C	TST (min)	432 (60.34)	430.66 (61.12)	16.27 + −0.03 x ref	bias − 2.46(16.02 + −0.02 x ref)	bias + 2.46(16.02 + −0.02 x ref)
ACC+T+HRV+C	Light (min)	249.31 (48.24)	247.31 (45.3)	58.1 + −0.23 x ref	bias − 65	bias + 65
ACC+T+HRV+C	Deep (min)	90.43 (35.54)	93.34 (34.19)	26.85 + −0.32 x ref	bias − 52.63	bias + 52.63
ACC+T+HRV+C	REM (min)	92.26 (26.19)	90.01 (26.24)	42.83 + −0.45 x ref	bias − 2.46(10.58 + 0.08 x ref)	bias + 2.46(10.58 + 0.08 x ref)

**Table 4:** Epoch by epoch sensitivity and specificity for wake, light, deep, and REM sleep and the four models compared in this paper.

Model	Stage	Accuracy	Sensitivity	Specificity
ACC	Wake	94.53 (3.79) [94.18, 94.88]	72.07 (17.61) [70.44, 73.74]	96.85 (2.94) [96.58, 97.14]
ACC	Light	61.27 (5.72) [60.74, 61.8]	67.74 (14.22) [66.45, 69.03]	53.11 (16.2) [51.63, 54.62]
ACC	Deep	80.44 (6.01) [79.87, 81]	48.14 (28.42) [45.51, 50.77]	87.15 (7.88) [86.42, 87.9]
ACC	REM	78.98 (5.2) [78.5, 79.47]	28.99 (19.37) [27.19, 30.81]	90.72 (5.89) [90.17, 91.27]
ACC+T	Wake	94.79 (3.93) [94.43, 95.17]	73.71 (17.9) [72.07, 75.41]	97.05 (2.79) [96.79, 97.32]
ACC+T	Light	63.09 (6.8) [62.46, 63.72]	70.86 (12) [69.76, 71.98]	53.77 (16.32) [52.27, 55.31]
ACC+T	Deep	82.69 (5.91) [82.14, 83.25]	50.06 (28.88) [47.33, 52.71]	89.67 (6.66) [89.07, 90.29]
ACC+T	REM	79.9 (5.35) [79.4, 80.4]	32.38 (19.33) [30.55, 34.18]	91.04 (5.44) [90.55, 91.57]
ACC+T+HRV	Wake	95.58 (3.5) [95.25, 95.92]	77.18 (16.77) [75.65, 78.76]	97.61 (2.11) [97.42, 97.81]
ACC+T+HRV	Light	77.48 (6.14) [76.91, 78.05]	79.13 (7.38) [78.45, 79.82]	75.73 (11.75) [74.62, 76.84]
ACC+T+HRV	Deep	89.11 (4.25) [88.72, 89.51]	69.57 (23.84) [67.39, 71.8]	93.73 (4.16) [93.35, 94.12]
ACC+T+HRV	REM	90.16 (4.18) [89.78, 90.57]	75.89 (18.09) [74.23, 77.56]	93.75 (3.4) [93.44, 94.06]
ACC+T+HRV+C	Wake	96.38 (3.12) [96.1, 96.68]	80.74 (14.12) [79.43, 82.06]	98.15 (1.87) [97.98, 98.33]
ACC+T+HRV+C	Light	80.2 (5.53) [79.68, 80.73]	81.7 (6.97) [81.06, 82.35]	78.67 (10.63) [77.7, 79.67]
ACC+T+HRV+C	Deep	90.64 (3.75) [90.29, 90.99]	74.44 (20.28) [72.56, 76.34]	94.63 (3.77) [94.28, 94.99]
ACC+T+HRV+C	REM	90.87 (4.12) [90.49, 91.26]	78.08 (17.39) [76.5, 79.71]	94.12 (3.41) [93.8, 94.44]

## Data Availability

The dataset supporting the conclusions of this article is not available due to privacy and ethical reasons.
